# Predictive analysis of all-cause mortality of previously untreated pulmonary tuberculosis patients complicated by hypertension

**DOI:** 10.3389/fcimb.2025.1574824

**Published:** 2025-08-11

**Authors:** Anhua Cao, You Nie, Zhun Zhong, Yi Pei, Ping Deng, Hebin Xie, Yiping Leng

**Affiliations:** ^1^ The Affiliated Changsha Central Hospital, Department of Cardiology, Hengyang Medical School, University of South China, Changsha, Hunan, China; ^2^ The Affiliated Changsha Central Hospital, Department of Laboratory Medicine, Hengyang Medical School, University of South China, Changsha, Hunan, China; ^3^ The Affiliated Changsha Central Hospital, Center of Tuberculosis Diagnosis and Treatment, Hengyang Medical School, University of South China, Changsha, Hunan, China; ^4^ The Affiliated Changsha Central Hospital, Technology Demonstration Base for Tuberculosis Diagnosis and Treatment in Hunan Province, University of South China, Changsha, Changsha, Hunan, China; ^5^ The Affiliated Changsha Central Hospital, Department of Science and Education, Hengyang Medical School, University of South China, Changsha, Hunan, China; ^6^ The Affiliated Changsha Central Hospital, Changsha Tuberculosis Research Institute, Hengyang Medical School, University of South China, Changsha, Hunan, China; ^7^ The Affiliated Changsha Central Hospital, Changsha Technology Innovation Center for Tuberculosis Diagnosis and Treatment, University of South China, Changsha, Hunan, China

**Keywords:** previously untreated pulmonary tuberculosis, hypertension, mortality rate, risk factors, prediction model

## Abstract

**Objective:**

To investigate the risk factors for all-cause mortality of previously untreated pulmonary tuberculosis patients complicated by hypertension and construct a predictive model.

**Methods:**

We retrospectively analyzed the clinical data of inpatients with previously untreated pulmonary tuberculosis complicated by hypertension from 2019 to 2021 in Changsha Central Hospital. Patients’ survival status and cardiovascular events were collected through telephone follow-up. LASSO regression was utilized to screen predictive variables, and binary logistic regression identified mortality risk factors. A predictive nomogram model was developed using R software, and its precision and reliability were verified.

**Results:**

Among the 1,014 patients, there were 100 (9.86%) deaths and 82 (8.09%) cardiovascular events. LASSO regression screened out 13 predictive variables. Multivariate logistic regression analysis revealed that smoking history, sputum bacteriology, pleural effusion, coronary heart disease, and chronic kidney disease were independent risk factors. Based on the training set data, a nomogram prognostic model was developed, showing an AUC of 0.712 (95% CI: 0.777-0.847), with 50.0% sensitivity and 84.3% specificity. The model’s fit was confirmed through internal and external validations.

**Conclusion:**

The prediction model constructed in this study has high predictive ability and satisfactory clinical efficacy, and can provide an effective individualized prediction tool for assessing all-cause mortality risk in patients with previously untreated pulmonary tuberculosis complicated by hypertension.

## Introduction

Tuberculosis (TB) is a long-term infectious disease caused by Mycobacterium tuberculosis (MTB), mainly spread via the respiratory tract. According to The World Health Organization’s (WHO) “Global Tuberculosis Report 2024” (World Health Organization, 2024), in 2023, there were about 10.8 million new TB cases globally, with an incidence rate of 134/100,000 and about 1.25 million fatal cases. Compared to 2015, the incidence and mortality rates of tuberculosis in 2023 have decreased by 8.3% and 23% respectively, which is far from reaching the second milestone goal set in the End TB Strategy, which aims to reduce the incidence by 50% and mortality by 75% by 2025. Identifying the risk factors for mortality in TB patients is crucial to reducing the burden and achieving the “End TB” goals.

The lung is the predominant site of tuberculosis infection, and pulmonary tuberculosis (PTB) is the most lethal variant of the disease. Meanwhile, previously untreated tuberculosis accounts for 80%-90% of the total number of newly diagnosed tuberculosis patients, and its drug resistance is relatively simple. In this context, some studies have analyzed the mortality causes of PTB patients and shown that the overall disease burden caused by TB can be attributed to post-TB sequelae (Menzies et al., 2021) or non-communicable diseases, such as respiratory diseases and cardiovascular diseases (Briggs et al., 2012). The latest meta-analysis evidence indicates that the all-cause mortality in patients treated for tuberculosis is largely attributed to cardiovascular diseases (20% [95% CI 15–26]; I²=92%) (Romanowski et al., 2019). A history of tuberculosis increases the risk of myocardial infarction, ischemic stroke, and peripheral arterial disease (Wang et al., 2017; Salindri et al., 2019). Recent literature reports that Mycobacterium bovis infection (BCG) can enhance atherosclerosis development (Huaman et al., 2020). A retrospective cohort study based on a health insurance research database in Taiwan, China (Chung et al., 2014) found that the proportion of hypertension in the pulmonary tuberculosis population was 38.7%, which was significantly higher than that in non-TB populations. Patients with PTB complicated by hypertension often have worse prognosis. The study by (Seegert et al., 2021). included 1,544 initial-treated PTB patients, in whom the proportion of those with hypertension was 12.8%. Compared with normotensive PTB patients, hypertensive PTB patients had a lower likelihood of successful treatment (odds ratio [OR] 0.76, 95% confidence interval [CI] 0.52-1.10) and a higher mortality rate during the two-year follow-up (hazard ratio [HR] 1.64, 95% CI 1.15-2.34). There is a relatively common comorbidity between pulmonary tuberculosis and hypertension, and the two diseases can interact with each other, increasing the death burden of each other.

In recent years, nomograms have been considered a feasible and effective predictive tool for disease diagnosis and prognosis outcome assessment (Jennings, 2015; Seegert et al., 2021). However, there has been no research on the prognosis of patients with a special condition of pulmonary tuberculosis combined with hypertension. The mycobacterial resistance in patients with primary-treated pulmonary tuberculosis is relatively simple, facilitating the evaluation of new therapeutic methods and enhancing the understanding of the disease’s natural course. This study was designed to delve into the risk factors influencing all-cause mortality among patients diagnosed with previously untreated pulmonary tuberculosis and hypertension, to create a nomogram prediction model applicable in clinical settings. The calibration and discrimination of the model were assessed to determine its effectiveness, which has good clinical value for evaluating clinical prognosis in the target population.

## Methods

### Study subject selection

Adult inpatients with previously untreated PTB complicated by hypertension in the Changsha Central Hospital Affiliated with the University of South China were enrolled as our primary cohort from 1st January, 2019 to 31st December, 2021. Patients in China receive treatment at designated facilities and obtain relevant insurance subsidies (Liu et al., 2020a). The Tuberculosis Department of Changsha Central Hospital, a specialized unit in Hunan Province, is among the facilities that admit and treat the highest volume of TB patients in the region. Consequently, the TB cases in Changsha Central Hospital provide a vital insight into the epidemiological trends and disease features prevalent in Hunan.

The research subjects were chosen according to the following criteria: 1) Initially treated PTB patients (Wu et al., 2024), which includes: (a) individuals who have never received anti-TB medications for PTB, (b) individuals who have not completed the full course of standard chemotherapy, and (c) individuals who have undergone irregular chemotherapy for less than one month; 2) Patients with essential hypertension; 3) individuals aged 18 years or older; 4) Patients who are not pregnant or lactating. 5) Patients without diseases that affect immune function, such as AIDS, malignant tumors, hematological diseases, systemic lupus erythematosus, rheumatoid arthritis, kidney transplantation, etc. 6) Patients had not used hormones and immunosuppressants within 4 months. 7) The clinical baseline data of patients were complete.

The study was conducted in accordance with the Declaration of Helsinki. It was a retrospective study that utilized patient data from the case management system, and ethical approval was obtained with waived informed consent. This ethical approval was granted by the Medical Ethics Sub-committee of Changsha Central Hospital (Ethics Approval Number: 2023-049).

### Data collection

We conducted a retrospective data collection from 1,014 hospitalised patients with PTB combined with hypertension using the medical record system of Changsha Central Hospital. The extracted data included: 1) General clinical data such as age, gender, time of admission, type of medical insurance, educational background, and the presence of comorbidities like diabetes, chronic kidney disease, coronary heart disease, heart failure, COPD, as well as smoking history, drinking history, blood pressure and heart rate measurements at admission, the grading of hypertension, the duration of PTB, sputum bacteriological findings (If either AFB smear or sputum culture is positive, the case is considered sputum bacteriologically positive), drug resistance, and medication usage patterns for antihypertensives and lipid-lowering drugs. 2) Laboratory test results comprised neutrophil, platelet (PLT), lymphocytes, hemoglobin (Hb), albumin, transaminase, creatinine, blood lipid, erythrocyte sedimentation rates (ESR), and C-reactive protein (CRP). 3) Imaging findings involved the presence of cavities, pleural effusion, and the number of lung lobes affected by the lesion. 4) Follow-up data included the course of anti-tuberculosis treatment and adherence to medication regimens.

### Statistical analysis

Statistical analyses were conducted using the R statistical package 4.3.2 (http://www.R-Project). Continuous measurements that followed a normal distribution were reported as the mean and standard deviation (SD), while those that did not conform to a normal distribution were presented as the median (IQR). Comparisons between the two groups were conducted using an independent samples t-test or the Mann-Whitney U test. Categorical variables were expressed as frequency percentages (%) and analyzed using the χ^2^ test. LASSO logistic regression analysis was employed to identify candidate predictors with substantial regression coefficients. Backward stepwise logistic regression was utilized for multivariate analysis to identify statistically significant prognostic risk factors in patients with previously untreated PTB complicated by hypertension. The model’s predictive performance was assessed through the receiver operating characteristic curve (ROC), calibration curve, and decision curve analysis (DCA). A confidence interval (CI) of 95% was established, and a p-value of <0.05 (bilateral) was deemed statistically significant.

## Results

### Traits of research subjects

**Figure 1:**
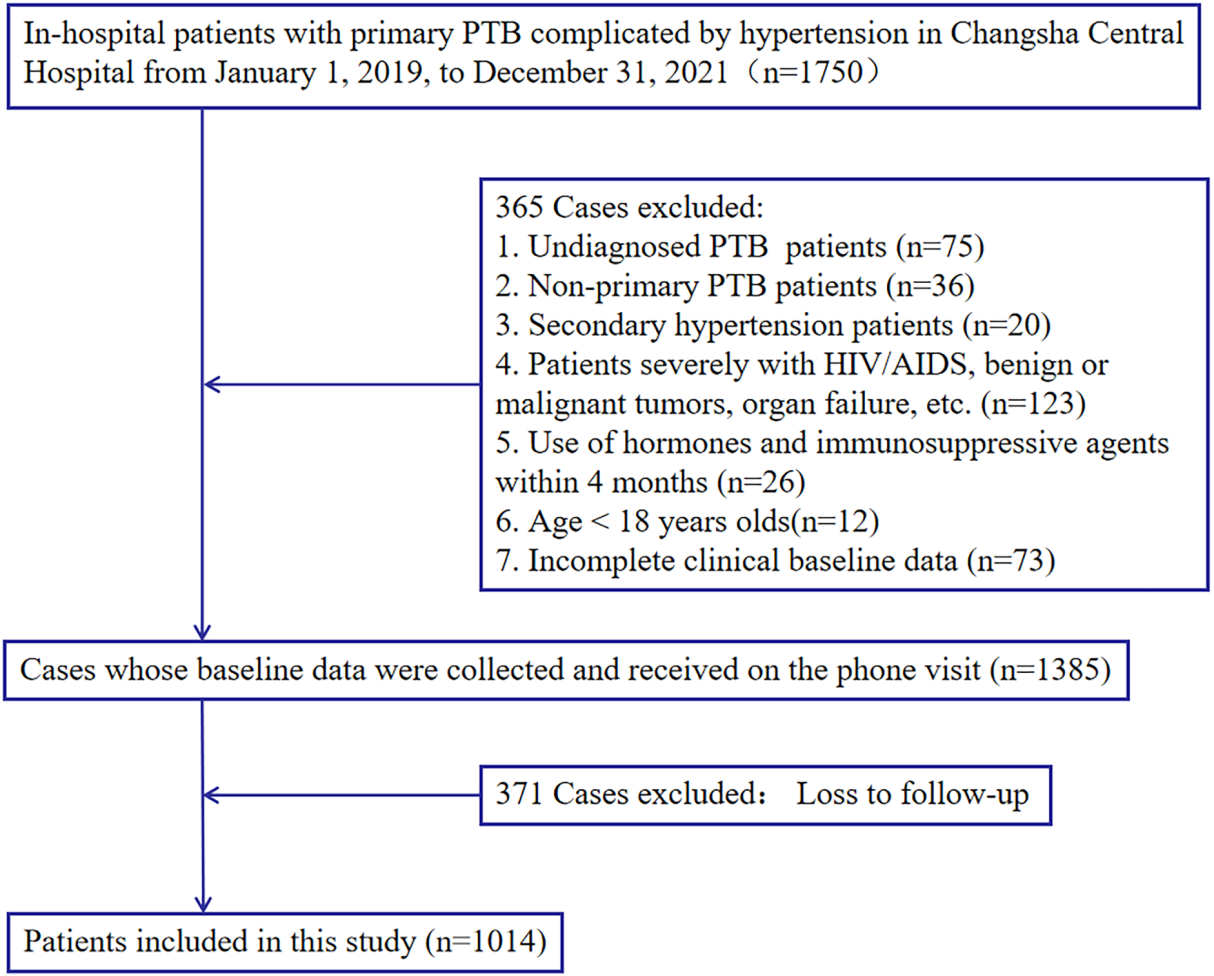
Study design. A total of 1,014 in-hospital patients with previously untreated PTB complicated by hypertension with complete relevant data were enrolled in this study.

The research included 1014 hospitalized patients with previously untreated PTB complicated by hypertension who were screened, as presented in **Figure 1**. The data was randomly partitioned into training (n=710) and validation(n=304) cohorts at a 7:3 ratio (random seed number: 20220326), with patients classified into death or survival groups based on all-cause mortality.

Among the 1,014 subjects, 100 patients died, with a mortality rate of 9.86%, and 82 patients (8.09%) had cardiovascular events. The study included patients with an average age of 67. The proportion of male patients (70.32%) exceeded that of female patients (20.68%). Patients with junior high school education or below accounted for 81.46%. Regarding insurance, 53.96% had urban coverage, while 41.22% had rural coverage. Smokers (47.44%) and drinkers (26.73%) accounted for relative proportions. The patients (51.68%) were positive for sputum bacteriology, 44.38% had extrapulmonary tuberculosis, 28.60% had cavitary manifestations on chest imaging, and 27.31% had pleural effusion manifestations on imaging. Patients with comorbidities accounted for 39.30% diabetes, 8.38% heart failure, 19.92% hyperlipidemia, 14.69%stroke, 27.91%coronary heart disease, 10.26% chronic kidney disease (CKD), and 10.95% chronic obstructive pulmonary disease (COPD). Patients (40.4%) had adverse drug reactions during the treatment of PTB. Patients (73.08%) took antihypertensive drugs, while 23.67% took lipid-lowering drugs. Supplementary baseline data is available in **Table 1**.

**Table 1:** Baseline characteristics of included in-hospital patients with previously untreated PTB complicated by hypertension.

Variable	Total data set (n=1014)	Training set (n=710)	Validation set (n=304)
Outcome (%)			
Survival	914 (90.14)	644 (90.70)	270 (88.82)
Death	100 (9.86)	66 (9.30)	34 (11.18)
Gender (%)			
Male	713 (70.32)	504 (70.99)	209 (68.75)
Female	301 (29.68)	206 (29.01)	95 (31.25)
Medical insurance (%)			
Urban	537 (52.96)	375 (52.82)	162 (53.29)
Rural	418 (41.22)	295 (41.55)	123 (40.46)
Other	59 (5.82)	40 (5.63)	19 (6.25)
Educational attainment (%)			
Junior middle school	826 (81.46)	560 (78.87)	266 (87.50)
Junior high school or above	188 (18.54)	150 (21.13)	38 (12.50)
Smoking (%)			
No	533 (52.56)	362 (50.99)	171 (56.25)
Yes	481 (47.44)	348 (49.01)	133 (43.75)
Drinking (%)			
No	743 (73.27)	509 (71.69)	234 (76.97)
Yes	271 (26.73)	201 (28.31)	70 (23.03)
Sputum bacteriology (%)			
No	490 (48.32)	341 (48.03)	149 (49.01)
Yes	524 (51.68)	369 (51.97)	155 (50.99)
Extrapulmonary tuberculosis (%)			
No	564 (55.62)	400 (56.34)	164 (53.95)
Yes	450 (44.38)	310 (43.66)	140 (46.05)
Cavity (%)			
No	724 (71.40)	502 (70.70)	222 (73.03)
Yes	290 (28.60)	208 (29.30)	82 (26.97)
Pleural effusion (%)			
No	737 (72.68)	515 (72.54)	222 (73.03)
Yes	277 (27.31)	195 (27.46)	82 (26.97)
Hypertension classification (%)			
1	126 (12.43)	97 (13.66)	29 (9.54)
2	326 (32.15)	235 (33.10)	91 (29.93)
3	562 (64.30)	378 (53.24)	184 (60.53)
Diabetes (%)			
No	618 (60.95)	431 (60.70)	187 (61.51)
Yes	397 (39.15)	279 (39.30)	117 (38.49)
Heart failure (%)			
No	929 (91.62)	635 (91.97)	276 (90.79)
Yes	85 (8.38)	57 (8.03)	28 (9.21)
Hyperlipidemia (%)			
No	812 (80.08)	572 (80.56)	240 (78.95)
Yes	202 (19.92)	138 (19.44)	64 (21.05)
Stroke (%)			
No	865 (85.31)	602 (84.79)	263 (86.51)
Yes	149 (14.69)	108 (15.21)	41 (13.49)
CHD (%)			
No	731 (72.09)	513 (72.25)	218 (71.71)
Yes	283 (27.91)	197 (27.75)	86 (28.29)
CKD (%)			
No	910 (89.74)	648 (91.27)	262 (86.18)
Yes	104 (10.26)	62 (8.73)	42 (13.82)
COPD (%)			
No	903 (89.05)	627 (88.31)	276 (90.79)
Yes	111 (10.95)	83 (11.69)	28 (9.21)
Adverse reactions to anti-TB drugs (%)			
No	604 (59.57)	414 (58.31)	190 (62.50)
Yes	410 (40.43)	296 (41.69)	114 (37.50)
Antihypertensive medications (%)			
No	273 (29.92)	207 (29.15)	66 (21.71)
Yes	741 (73.08)	503 (70.85)	238 (78.29)
Lipid lowering drugs (%)			
No	774 (76.33)	544 (76.62)	230 (75.66)
Yes	240 (23.67)	166 (23.38)	74 (24.34)
Age (years)	67.00 [58.00, 74.00]	67.00 [59.00, 75.00]	67.00 [58.00, 74.00]
Hypertension course (years)	6.00 [2.00, 10.00]	6.00 [2.00, 10.00]	6.00 [2.00, 10.00]
Lobe count (lobes)	4.00 [2.00, 6.00]	4.00 [2.00, 6.00]	4.00 [2.00, 6.00]
Course of anti-TB treatment (months)	12.00 [6.00, 12.00]	12.00 [6.00, 12.00]	12.00 [8.00, 12.00]
WBC (X10^9^/L)	6.26 [4.95, 8.14]	6.29 [4.90, 8.26]	6.23 [5.04, 8.01]
PLT (X10^9^/L)	234.00 [180.00, 296.0]	236.00 [181.50, 294.80]	229.00 [176.00, 300.00]
HGB (g/L)	119.00 [103.00, 132.00]	120.00 [104.20, 133.80]	116.00 [100.00, 130.00]
Cr (μmol/L)	69.00 [56.00, 91.00]	68.00 [55.00, 89.00]	70.00 [58.00, 93.25]
ALB (g/L)	35.00 [30.00, 39.00]	35.00 [30.00, 39.00]	35.00 [30.00, 39.00]
AST (U/L)	21.00 [17.00, 29.00]	21.50 [17.00, 29.00]	21.00 [17.00, 28.00]
ALT (U/L)	15.00 [10.00, 25.00]	15.00 [10.00, 25.00]	14.00 [10.00, 25.00]
TC (mmol/L)	4.14 [3.49, 4.84]	4.12 [3.50, 4.79]	4.26 [3.46, 4.98]
LDL-C (mmol/L)	2.44 [1.93, 3.10]	2.39 [1.94, 3.04]	2.50 [1.90, 3.14]
HDL-C (mmol/L)	1.00 [0.82, 1.22]	1.00 [0.82, 1.23]	1.00 [0.81, 1.22]
CRP (mg/L)	9.68 [3.14, 44.60]	11.35 [3.14, 44.58]	9.31 [3.27, 44.65]
ESR (mm/h)	36.00 [17.00, 65.00]	36.00 [17.00, 64.75]	38.00 [19.00, 68.50]

CHD, Coronary heart disease; CKD, Chronic Kidney Disease; COPD, Chronic Obstructive Pulmonary Disease; WBC, white blood cell; PLT, platelet; HGB, Hemoglobin; Cr, creatinine; ALB, Albumin; AST, aspartate transaminase; ALT, Alanine aminotransferase; TC, Serum total cholesterol; LDL-C, Low-Density Lipoprotein Cholesterol; HDL-C, High-density lipoprotein cholesterol; CRP, C-reactive protein; ESR, erythrocyte sedimentation rate.

Among 1,014 research subjects, 82 patients had cardiovascular events, including 5 cases (6.10%) of myocardial infarction, 4 cases (4.88%) undergoing coronary interventional therapy, 9 cases (10.98%) of stroke, 25 cases (45.12%) of heart failure, 37 cases (45.12%) of cardiogenic death, and 2 cases (2.44%) of macrovascular lesions. (**Table 2**).

**Table 2:** Distribution and proportion of cardiovascular events.

Cardiovascular events	Total (n=82)	Training set (n=58)	Validation set (n=24)
Myocardial Infarction (%)	5 (6.10)	3 (5.17)	2 (8.33)
Coronary Intervention Therapy (%)	4 (4.88)	3 (5.17)	1 (4.17)
Stroke (%)	9 (10.98)	6 (10.34)	3 (12.50)
Heart Failure (%)	25 (30.59)	18 (31.03)	7 (29.17)
Cardiac Death (%)	37 (45.12)	26 (44.83)	11 (45.83)
Macrovascular Disease (%)	2 (2.44)	2 (0.34)	0 (0)

The 710 patients in the training set were divided into death and survival groups. The findings indicated statistically significant differences between the two groups regarding age, sputum bacteriology, pleural effusion, heart failure, stroke, CHD, CKD, number of lung lobes, anti-tuberculosis treatment, WBC, Cr, ESR, CRP, Hb, Alb, total cholesterol, and LDL (P < 0.05), as illustrated in **Table 3**.

**Table 3:** Baseline data of the death group and the survival group in the training set.

Variables	Survival group (n=644)	Death group (n=66)	P
Gender (%)			
Male	193 (29.97)	13 (19.70)	0.08
Female	451 (70.03)	53 (80.30)	
Medical insurance (%)			
Urban	35 (5.43)	5 (7.58)	0.225
Rural	274 (42.55)	21 (31.82)	
Other	335 (52.02)	40 (60.61)	
Educational attainment (%)			
junior middle school	142 (22.05)	8 (12.12)	0.06
Junior high school or above	502 (77.95)	58 (87.88)	
Smoking (%)			
No	335 (52.02)	27 (40.91)	0.086
Yes	309 (47.98)	39 (59.09)	
Drinking (%)			
No	462 (71.74)	47 (71.21)	0.928
Yes	182 (28.26)	19 (28.79)	
sputum bacteriology (%)			
negative	320 (49.69)	21 (31.82)	0.006
positive	324 (50.31)	45 (68.18)	
Extrapulmonary tuberculosis (%)			
No	366 (56.83)	34 (51.52)	0.407
Yes	278 (43.17)	32 (48.48)	
Cavity (%)			
No	459 (71.27)	43 (65.15)	0.298
Yes	185 (28.73)	23 (34.85)	
Pleural effusion (%)			
No	481 (74.69)	34 (51.52)	<0.001
Yes	163 (25.31)	32 (48.48)	
Hypertension classification (%)			
1	90 (13.98)	7 (10.61)	0.313
2	217 (33.70)	18 (27.27)	
3	337 (52.33)	41 (62.12)	
Diabetes (%)			
No	394 (61.18)	37 (56.06)	0.417
Yes	250 (38.82)	29 (43.94)	
Heart failure (%)			
No	597 (92.70)	56 (84.85)	0.025
Yes	47 (7.30)	10 (15.15)	
Hyperlipidemia (%)			
No	521 (80.90)	51 (77.27)	0.478
Yes	123 (19.10)	15 (22.73)	
Stroke (%)			
No	554 (86.02)	48 (72.73)	0.004
Yes	90 (13.98)	18 (27.27)	
CHD (%)			
No	476 (73.91)	37 (56.06)	0.002
Yes	168 (26.09)	29 (43.94)	
CKD (%)			
No	596 (92.55)	52 (78.79)	<0.001
Yes	48 (7.45)	14 (21.21)	
COPD (%)			
No	571 (88.66)	56 (84.85)	0.358
Yes	73 (11.34)	10 (15.15)	
Adverse reactions to anti-TB drugs (%)			
No	374 (58.07)	40 (60.61)	0.691
Yes	270 (41.93)	26 (39.39)	
Antihypertensive medications (%)			
No	189 (29.35)	18 (27.27)	0.724
Yes	455 (70.65)	48 (72.73)	
Lipid lowering drugs (%)			
No	500 (77.64)	44 (66.67)	0.045
Yes	144 (22.36)	22 (33.33)	
Age (years)	67.00 [58.00, 74.00]	75.00 [68.00,80.00]	<0.001
Hypertension course (years)	5.00 [2.00, 10.00]	10.00 [2.25, 10.00]	0.131
Lobe count (lobes)	3.00 [2.00, 6.00]	5.00 [4.00, 6.00]	<0.001
Course of anti-TB treatment (months)	12.00 [7.00, 12.00]	7.50 [2.00, 12.00]	<0.001
WBC (X10^9^/L)	6.24 [4.89, 8.12]	7.68 [5.61, 9.41]	0.002
PLT (X10^9^/L)	236.00 [185.00,296.25]	220.00 [159.50,278.75]	0.093
HGB (g/L)	121.00 [107.00, 135.00]	101.00 [86.25,123.00]	<0.001
Cr (μmol/L)	67.00 [55.00, 85.00]	85.00 [64.00, 66.50]	<0.001
ALB (g/L)	35.50 [31.00, 39.00]	29.00 [26.00, 33.00]	<0.001
AST (U/L)	21.00 [17.00, 28.00]	23.00 [17.25, 34.25]	0.231
ALT (U/L)	15.00 [10.00, 25.00]	13.00 [9.00, 22.00]	0.142
TC (mmol/L)	4.14 [3.56, 4.80]	3.75 [3.03, 4.69]	0.019
LDL-C (mmol/L)	2.44 [1.95, 3.06]	2.20 [1.66, 2.87]	0.026
HDL-C (mmol/L)	1.01 [0.83, 1.23]	0.92 [0.72, 1.21]	0.083
CRP (mg/L)	10.35 [3.14, 40.43]	36.05 [7.41, 88.70]	<0.001
ESR (mm/h)	34.00 [16.00, 61.00]	58.50 [35.00, 88.50]	<0.001

CHD, Coronary heart disease; CKD, Chronic Kidney Disease; COPD, Chronic Obstructive Pulmonary Disease; WBC, white blood cell; PLT, platelet; HGB, Hemoglobin; Cr, creatinine; ALB, Albumin; AST, aspartate transaminase; ALT, Alanine aminotransferase; TC, Serum total cholesterol; LDL-C, Low-Density Lipoprotein Cholesterol; HDL-C, High-density lipoprotein cholesterol; CRP, C-reactive protein; ESR, erythrocyte sedimentation rate; p<0.05, statistically significant.

### Risk predictors for in-hospital patients initially diagnosed with primary PTB complicated by hypertension

**Figure 2:**
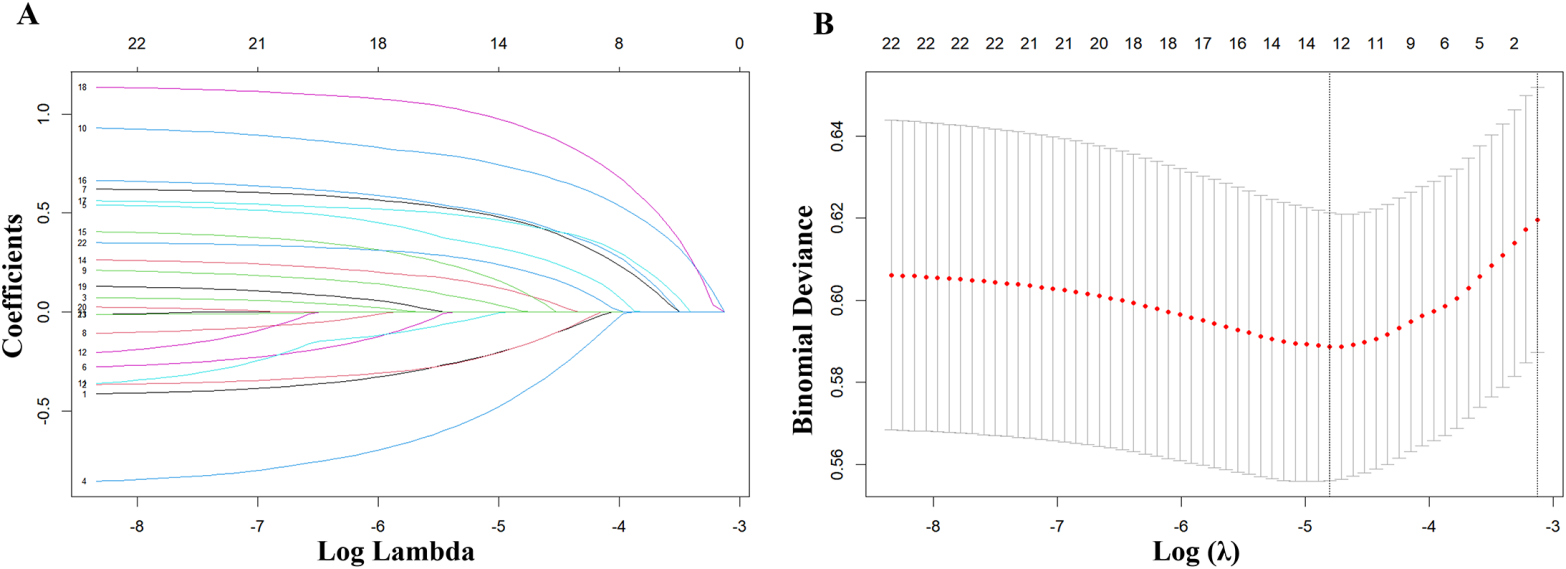
Lasso coefficient profile diagram **(A)** and Cross-validation plot **(B)**.

The LASSO regression analysis was used to screen out 13 predictive variables with non-zero coefficients, including gender, medical insurance, educational attainment, smoking history, sputum bacteriology, cavity, pleural effusion, heart failure, hyperlipidemia, stroke, coronary heart disease, chronic kidney disease, and taking lipid-lowering drugs. The LASSO regression analysis’s lasso coefficient profile diagram (**Figure 2A**) and cross-validation diagram (**Figure 2B**) were drawn.

**Figure 3:**
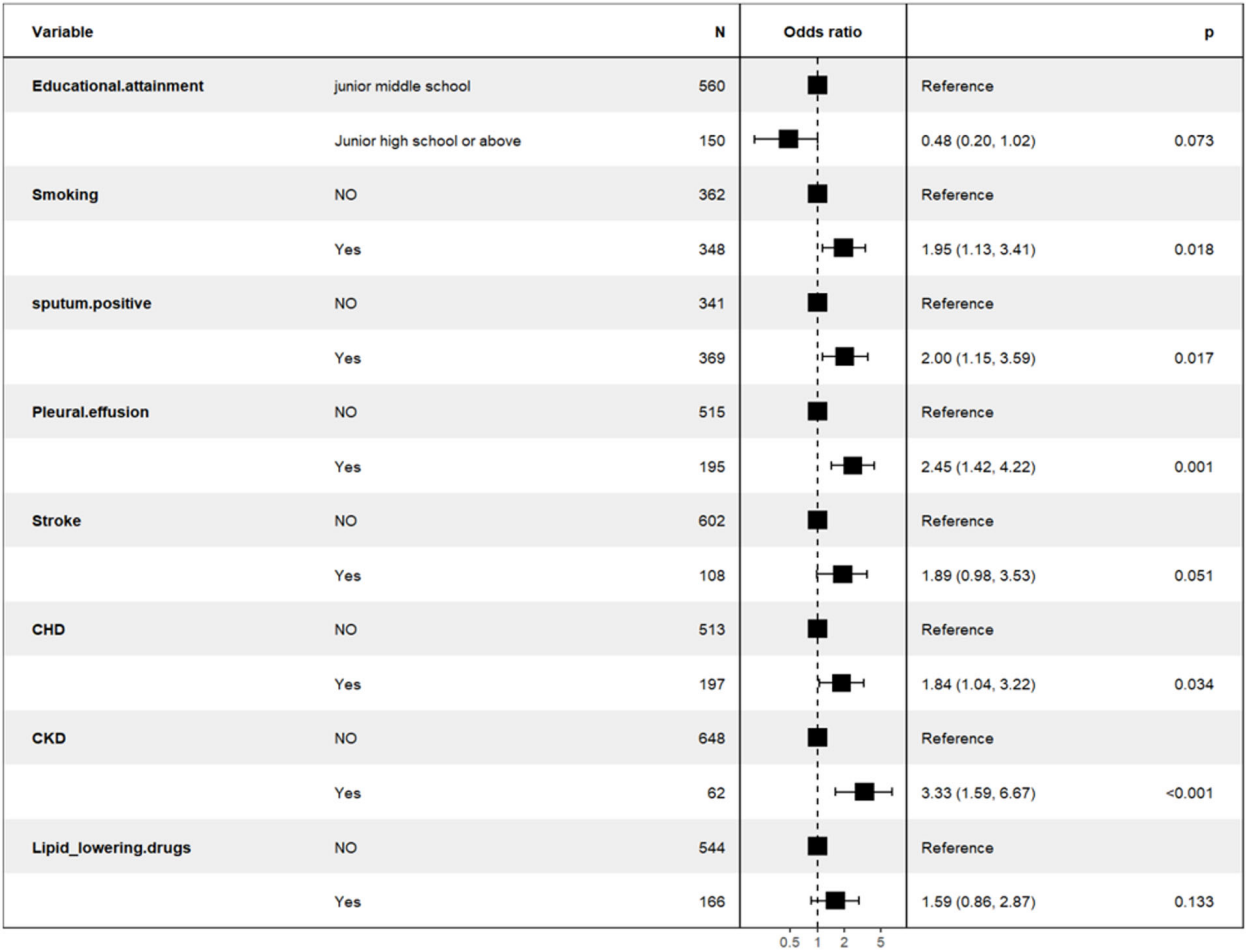
Backward stepwise logistic regression analysis of the training set of the population of in-hospital patients with previously untreated PTB complicated by hypertension.

Predictors demonstrating statistical significance (p<0.05) in preliminary analyses were subsequently included in the multivariate logistic regression model construction. The data presented in **Table 4** and **Figure 3** showed that smoking history (OR = 1.95; 95% CI: 1.13 - 3.41; p= 0.018), sputum bacteriology (OR = 2.00; 95% CI: 1.15 - 3.59; p = 0.017), pleural effusion (OR = 2.45; 95% CI: 1.42 - 4.22; p = 0.01), coronary heart disease (OR = 1.84; 95% CI: 1.04 - 3.22; p = 0.034), and chronic kidney disease (OR = 3.33; 95% CI: 1.59 - 6.67; p < 0.01) were identified as independent risk factors for mortality in patients with previously untreated PTB complicated by hypertension. (p < 0.05).

**Table 4:** Multivariate logistic regression analysis in the training set of in-hospital patients with previously untreated PTB complicated by hypertension.

Variable	OR	95% CI	P
Medical Insurance Type	2.08	0.99, 4.97	0.073
Smoking History	1.95	1.13, 3.41	0.018
Sputum Bacteriology	2.00	1.15, 3.59	0.017
Pleural Effusion	2.45	1.42, 4.22	0.001
Stroke	1.89	0.98, 3.53	0.051
Coronary Heart Disease	1.84	1.04, 3.22	0.034
Chronic Kidney Disease	3.33	1.59, 6.67	<0.001
Taking Lipid-lowering Drugs	1.59	0.86, 2.87	0.133

CI, confidence interval; OR, odds ratio; p < 0.05.

**Figure 4:**
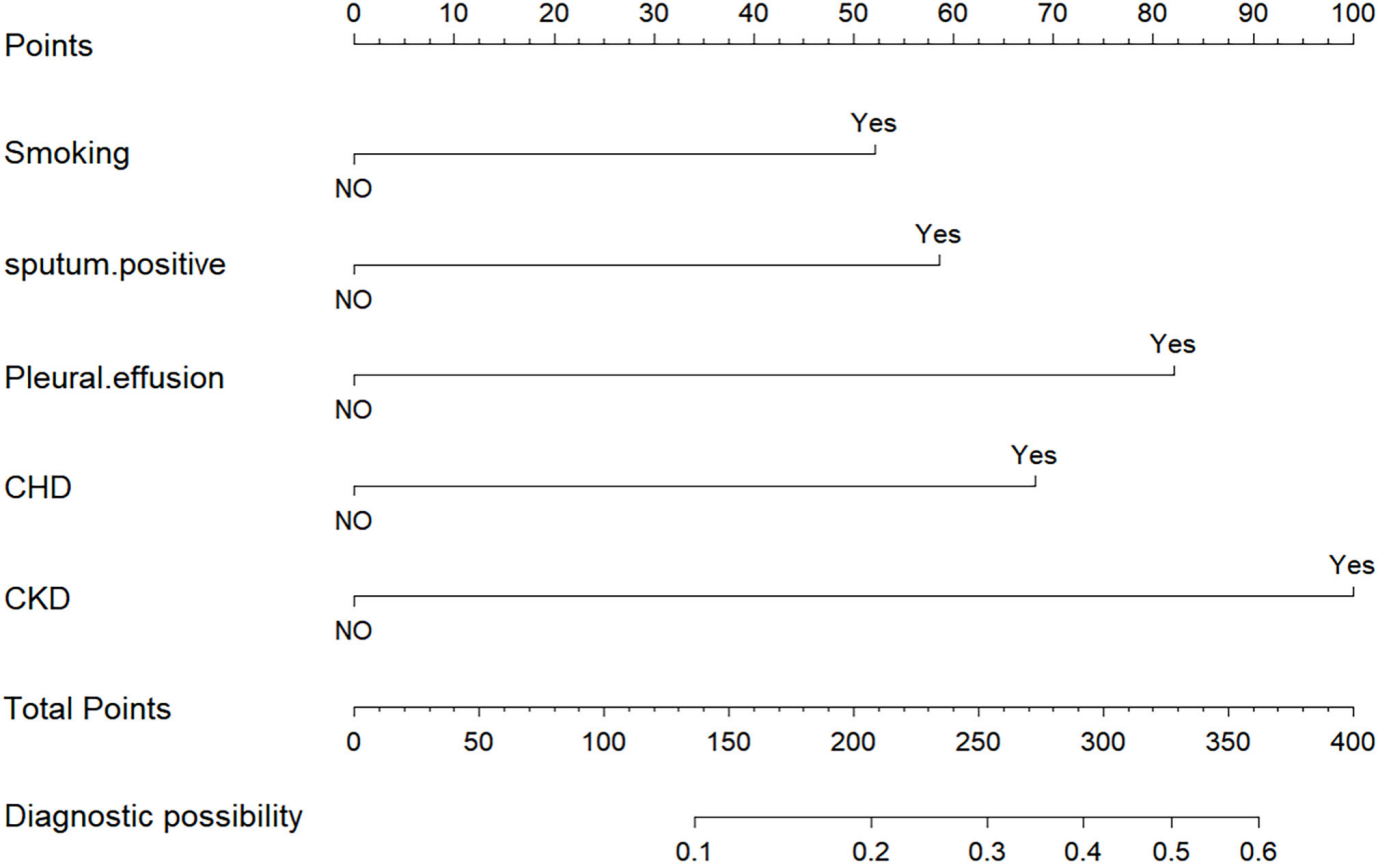
Nomogram to predict the outcomes of patients with previously untreated PTB complicated by hypertension.

A nomogram prognostic model was developed utilizing R software, incorporating five independent risk factors: smoking history, sputum bacteriology, pleural effusion, coronary heart disease, and chronic kidney disease, as shown in **Figure 4**.

**Figure 5:**
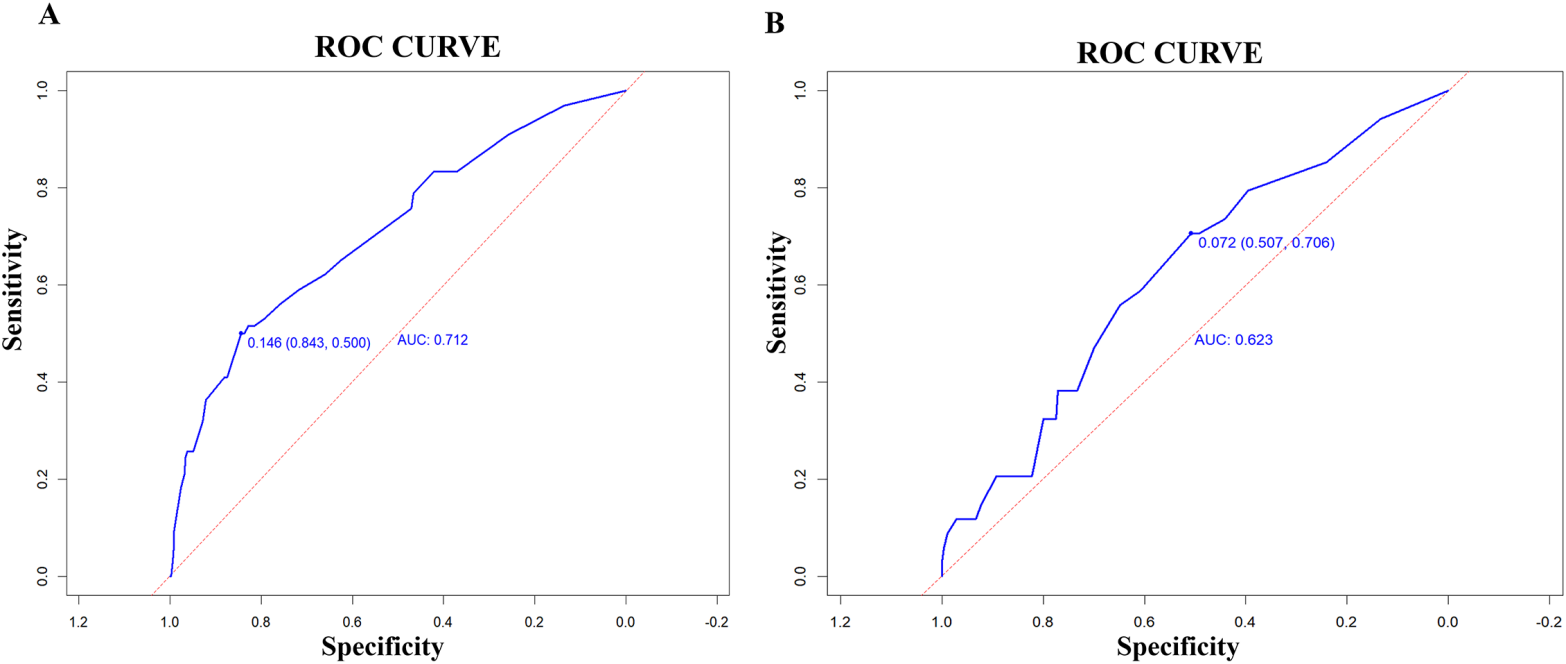
Calibration curves of the nomogram in the training set **(A)** and validation set **(B)**.

**Figure 6:**
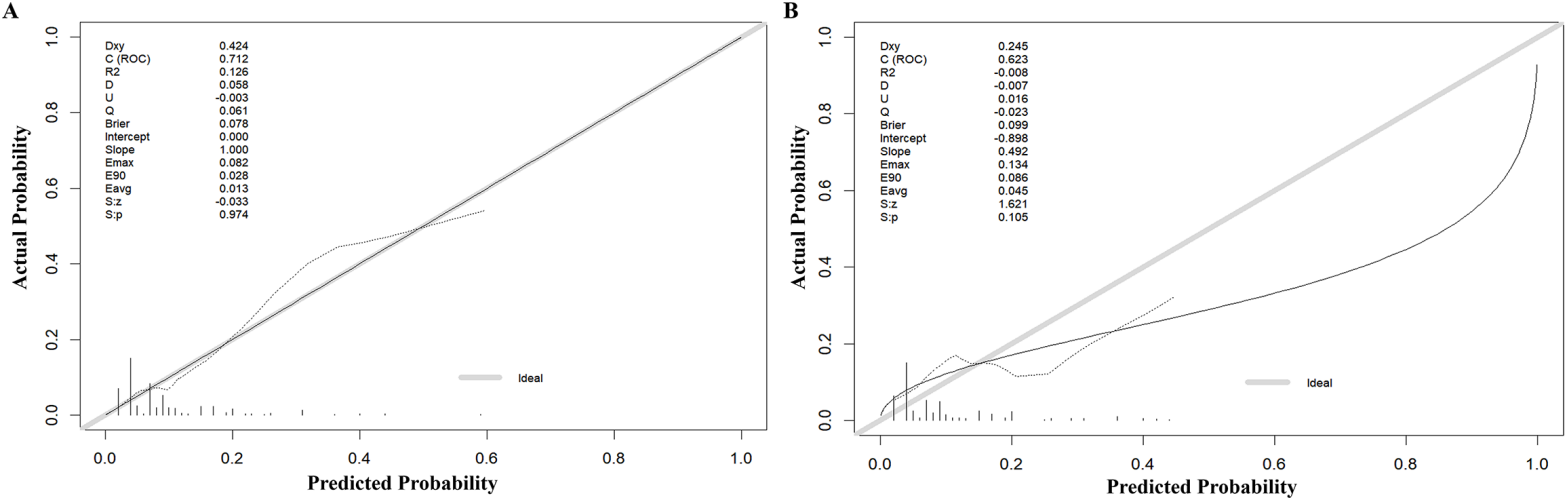
Calibration of the nomogram to predict the death in the training set **(A)** and validation set **(B)**.

**Figure 7:**
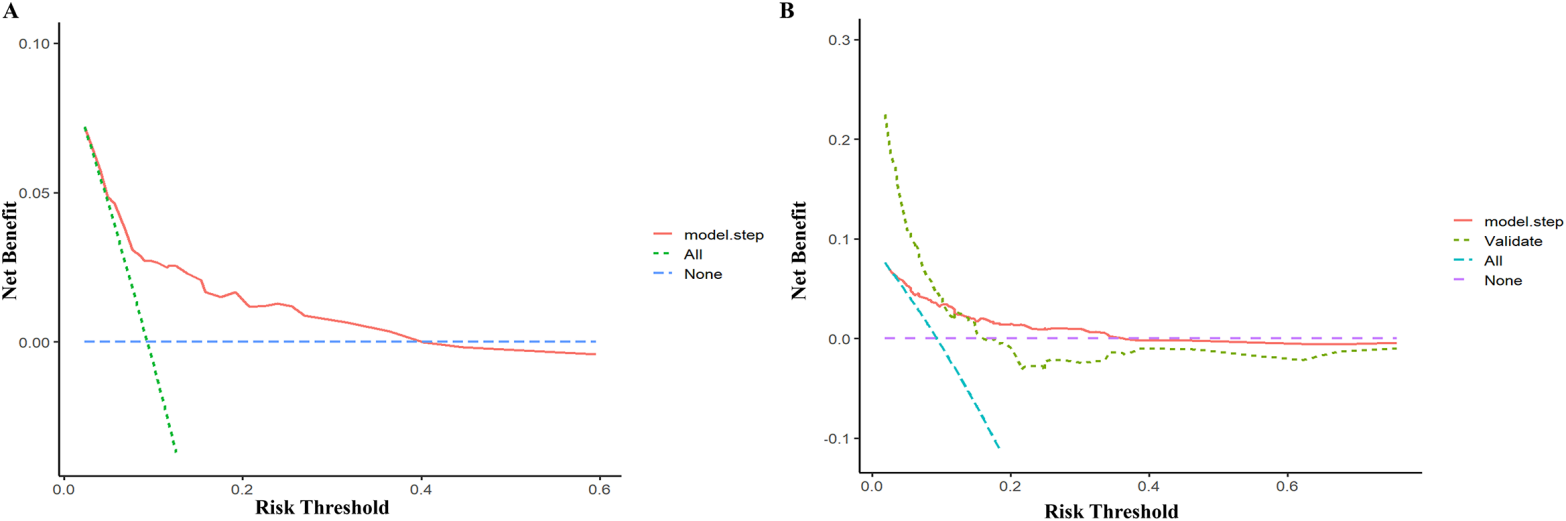
Decision cure analysis (DCA) for the nomogram in the training set **(A)** and validation set **(B)**.

The model’s discriminatory performance was quantified using ROC curve analysis, yielding an AUC of 0.712 in the training cohort with corresponding sensitivity and specificity values of 50.0% and 84.3%, respectively (**Figure 5A**). External validation results showed moderately reduced performance, with an AUC of 0.620 accompanied by increased sensitivity (70.6%) but decreased specificity (50.7%) in the independent validation cohort (**Figure 5B**). To evaluate the model’s predictive accuracy, calibration plots were created using R software for both the training (**Figure 6A**) and validation (**Figure 6B**) cohorts. These plots feature a horizontal axis representing the predicted mortality risk for hospitalized patients with previously untreated PTB complicated by hypertension, while the vertical axis displays the observed mortality rates within this patient population. The Brier score (the mean squared error between anticipated and actual values) of the calibration curve of the training set was 0.078, indicating a relatively good calibration effect. The S:p value was 0.974, indicating that the fitted line closely aligned with the standard reference line and possessed a rather good predictive value. The P-value of the Hosmer-Lemeshow test was 0.89, indicating that there was no significant difference between the predicted probabilities and the actual probabilities. In addition, R software was used to draw the clinical decision curves (DCA curves) of the training set (**Figure 7A**) and the validation set (**Figure 7B**). In the training set, when the threshold probability was between 5% and 40%, the decision curve was located above the “None” line and the “All” line, indicating the model’s clinical applicability within this interval. and the model had clinical practicability within this range. Similarly, in the validation set, when the threshold probability was between 5% and 20%, the model had clinical practicability.

## Discussion

China, as a nation with a significant tuberculosis burden, has experienced a gradual decrease in the mortality rate of the disease, however, the burden of death remains heavy (World Health Organization, 2024). As social and economic conditions improve and the population ages, the number of PTB patients with chronic non-communicable diseases like hypertension is increasing, further raising PTB mortality. Therefore, constructing a predictive model for the all-cause mortality risk in patients with previously untreated PTB complicated by hypertension is of great significance for improving patient prognosis.

The study’s target population comprised 1,014 patients with previously untreated PTB complicated by hypertension. The mortality rate was 9.86%, which is higher than the 4.9% mortality rate of patients with previously untreated PTB by Dan Li et al (Li et al., 2023), indicating that the complication of hypertension elevated the mortality risk for individuals with initially treated PTB to a significant degree. In another retrospective cohort study (Seegert et al., 2021), the treatment outcomes and prognoses of 1,544 individuals with previously untreated pulmonary tuberculosis (PTB) were followed up. The mortality rate of patients with previously untreated PTB complicated by hypertension within two years was significantly higher than those with normal blood pressure, further explaining this difference.

PTB and hypertension interact, increasing each other’s risk and severity, leading to a poorer prognosis. PTB can cause hypertension and cardiovascular diseases through chronic inflammation, immune responses (Caillon and Schiffrin, 2016; Rodriguez-Iturbe et al., 2017), and autoimmune reactions triggered by heat shock proteins (HSP) (Hazra et al., 2023). M. tuberculosis may exacerbate hypertension through lung tissue destruction and vascular inflammation (Kalla et al., 2020). Antituberculosis drugs like rifampicin (Liu et al., 2020b) may reduce the effectiveness of antihypertensive drugs. Hyperuricemia, a side effect of pyrazinamide (Yanai et al., 2021; Tian et al., 2023), increases cardiovascular disease risk. Hypertension promotes PTB progression through inflammatory factors and oxidative stress (Caillon and Schiffrin, 2016). Angiotensin II-induced hypertension may relate to TB severity (Cho et al., 2021). It is crucial to predict the mortality risk of patients with PTB complicated by hypertension in advance and take effective treatments promptly to improve the quality of life of patients.

This study created and validated a predictive nomogram model to assess the mortality risk of patients with previously untreated PTB complicated by hypertension. Eventually, five independent risk factors, namely smoking history, sputum bacteriology, pleural effusion, coronary heart disease, and chronic kidney disease, were screened out. Previous studies have found that age, male gender, smoking (Smith et al., 2015), sputum bacteriology (Wu et al., 2014; Liu et al., 2022), pleural effusion (Yen et al., 2017), combined chronic kidney disease, and malignant tumors are risk factors for the mortality of patients with PTB, which are consistent with the results of our study. In addition, we have newly discovered that combined coronary heart disease is an independent risk factor for them. A large amount of evidence-based medical evidence (Poole-Wilson et al., 2004; Jennings, 2015) has already shown that coronary heart disease increases the mortality risk.

Tuberculosis is linked to a heightened risk of developing atherosclerotic cardiovascular disease. Mycobacterial infection has been shown to aggravate atherosclerosis progression in mouse models lacking the low-density lipoprotein receptor (Ldlr-/-) (Huaman et al., 2020). In addition, hypertension accelerates the occurrence and development of coronary heart disease by inducing damage to vascular endothelial cells (Evans et al., 2021), promoting lipid deposition and platelet aggregation (D'Agostino et al., 2021), and increasing cardiac afterload (Hamada et al., 2022). When tuberculosis infection and hypertension coexist, the risk of coronary heart disease in patients increases sharply.

The AUC of the model was 0.712, indicating moderate predictive performance, with diagnostic sensitivity and specificity rates of 50.0% and 84.3%, respectively. A calibration curve assesses a model’s calibration performance by graphically comparing the predicted probabilities of outcomes against the empirically observed event frequencies. In this study, the predicted probabilities of the model were highly consistent with the actual probabilities, indicating that the model exhibits high accuracy. DCA evaluates the clinical utility of a model by quantifying its net benefit across varying threshold probabilities. In this study, DCA showed that the model provided clinical net benefit in the training set at threshold probabilities of 5%–40% and in the validation set at 5%–20%, indicating its clinical applicability within these ranges.

### Study limitations

This study is not without its limitations. To begin with, being a retrospective study, it is susceptible to residual confounding factors and selection bias. Moreover, the sample size for positive outcome events was small, and the external validation cohort was sourced from a single hospital, failing to adequately represent the diverse characteristics of the entire patient population, potentially leading to inaccurate predictions when applied in different settings. Additionally, certain variables had to be excluded from the study due to missing data. Many cases were lost to follow - up owing to telephone follow - up, potentially compromising the model’s accuracy. Forthermore, the relatively low sensitivity in this study may pose a risk of misjudging high-risk patients. Finally, the clinically relevant data and experimental parameters were merely a portion of the patient’s clinical data and may have omitted other significant data. Future research should optimize the model by integrating imaging/genetic markers to enhance accuracy, conduct multi-center external validation with diverse patient cohorts from different regions to assess generalizability and enhance representativeness, use standardized questionnaires (demographics, lifestyle, comorbidities) for comprehensive bias control, and incorporate TB-cardiovascular comorbidity biomarkers to refine predictive performance. This approach will enable rigorous assessment of the model’s generalizability and clinical utility across populations.

## Conclusions

Patients with previously untreated PTB complicated by hypertension have a relatively high mortality risk. The constructed nomogram prognostic model in this study may provide a feasible early assessment approach for the prognosis of patients with previously untreated PTB complicated by hypertension in clinical practice.

## Data Availability

The original contributions presented in the study are included in the article/**Supplementary Material**. Further inquiries can be directed to the corresponding authors.
